# Evidence of two distinct functionally specialized fibroblast lineages in breast stroma

**DOI:** 10.1186/s13058-016-0769-2

**Published:** 2016-11-03

**Authors:** Mikkel Morsing, Marie Christine Klitgaard, Abbas Jafari, René Villadsen, Moustapha Kassem, Ole William Petersen, Lone Rønnov-Jessen

**Affiliations:** 1 Department of Cellular and Molecular Medicine, University of Copenhagen, Copenhagen, Denmark; 2 Danish Stem Cell Centre, University of Copenhagen, Copenhagen, Denmark; 3 Department of Biology, University of Copenhagen, Copenhagen, Denmark; 4 Laboratory of Molecular Endocrinology, KMEB, Department of Endocrinology, Odense University Hospital and University of Southern Denmark, Odense, Denmark

**Keywords:** Breast, Epithelial morphogenesis, Fibroblasts, Mesenchymal stem cells

## Abstract

**Background:**

The terminal duct lobular unit (TDLU) is the most dynamic structure in the human breast and the putative site of origin of human breast cancer. Although stromal cells contribute to a specialized microenvironment in many organs, this component remains largely understudied in the human breast. We here demonstrate the impact on epithelium of two lineages of breast stromal fibroblasts, one of which accumulates in the TDLU while the other resides outside the TDLU in the interlobular stroma.

**Methods:**

The two lineages are prospectively isolated by fluorescence activated cell sorting (FACS) based on different expression levels of CD105 and CD26. The characteristics of the two fibroblast lineages are assessed by immunocytochemical staining and gene expression analysis. The differentiation capacity of the two fibroblast populations is determined by exposure to specific differentiating conditions followed by analysis of adipogenic and osteogenic differentiation. To test whether the two fibroblast lineages are functionally imprinted by their site of origin, single cell sorted CD271^low^/MUC1^high^ normal breast luminal epithelial cells are plated on fibroblast feeders for the observation of morphological development. Epithelial structure formation and polarization is shown by immunofluorescence and digitalized quantification of immunoperoxidase-stained cultures.

**Results:**

Lobular fibroblasts are CD105^high^/CD26^low^ while interlobular fibroblasts are CD105^low^/CD26^high^. Once isolated the two lineages remain phenotypically stable and functionally distinct in culture. Lobular fibroblasts have properties in common with bone marrow derived mesenchymal stem cells and they specifically convey growth and branching morphogenesis of epithelial progenitors.

**Conclusions:**

Two distinct functionally specialized fibroblast lineages exist in the normal human breast, of which the lobular fibroblasts have properties in common with mesenchymal stem cells and support epithelial growth and morphogenesis. We propose that lobular fibroblasts constitute a specialized microenvironment for human breast luminal epithelial progenitors, i.e. the putative precursors of breast cancer.

**Electronic supplementary material:**

The online version of this article (doi:10.1186/s13058-016-0769-2) contains supplementary material, which is available to authorized users.

## Background

Development of glandular organs such as the breast involves the process of branching morphogenesis, which is the result of an interaction between the epithelium and the surrounding mesenchyme [1]. Unlike most parenchymal epithelial cells, in general mesenchymal stromal cells have not been classified into particular lineages or considered participants of tissue specific stem cell hierarchies. However, evidence is accumulating that epithelial stem or progenitor competence is linked to proximity to specialized fibroblasts [2]. It is well-established that the human breast is characterized by the presence of two anatomically distinct types of stroma, a relatively less cellular, fibrous stroma embedding the interlobular ducts and a more cellular, loosely arranged stroma embedding the terminal ductules, *in toto* referred to as the TDLU [3, 4]. Under resting, homeostasis conditions the vast majority of cellular turnover takes place in TDLUs and is fuelled by cycling cells within the luminal epithelial lineage [5]. As the majority of breast cancer is also luminal and originates in TDLUs, the question of whether the stromal microenvironment contributes to cellular turnover in this compartment deserves some attention. As described here, our efforts to address this have led to the discovery of CD105^high^/CD26^low^ lobular fibroblasts which compared to CD105^low^/CD26^high^ interlobular fibroblasts resemble mesenchymal stem cells and support luminal epithelial growth and branching morphogenesis.

## Methods

### Tissue

Normal breast biopsies of which some were included in previous work [6] were collected with consent from women undergoing reduction mammoplasty for cosmetic reasons. The use and storage of human material has been approved by the Regional Scientific Ethical Committees (Region Hovedstaden, H-2-2011-052) and the Danish Data Protection Agency (2011-41-6722). Tissue samples for immunohistochemical staining were kept at −80 °C and epithelial organoids and fibroblasts were isolated as described [6, 7].

### Cell culture

Fibroblasts were plated in Primaria™ T-25 flasks (Becton Dickenson) [7] in DMEM/F-12 (DMEM:Ham’s F12 Nutrient Mixture (F12), 1:1 v/v, Life Technologies), with 2 mM glutamine and 1 % fetal bovine serum (FBS, Sigma). The cultures were split at a 1:3 ratio and expanded until the fourth to the fifth passage in collagen-coated flasks (Nunc, 8 μg collagen/cm^2^, PureColl, CellSystems) in basal medium with 5 % FBS prior to fluorescence activated cell sorting (FACS). Sorted fibroblasts were sub-cultured under the same conditions. Profiling of fibroblasts in the second and third passages from two biopsies, which had undergone limited, if any, proliferation [7] (plated on Primaria™ with 1 % FBS and switched to 5 % FBS upon passage) were included to ensure that the observed phenotypes represented primary cells. For comparison with breast fibroblasts a human telomerase, reverse transcriptase-immortalized, human mesenchymal stem cell (hMSC) line was employed [8].

### Flow cytometric analysis and FACS

Epithelial organoids or fibroblasts derived from a total of 13 biopsies were prepared for FACS as described [6]. To isolate CD271 (nerve growth factor receptor)^low^/mucin 1 (MUC1)^high^, luminal epithelial cells, suspended cells from organoids were incubated for 30 minutes at 4 °C in the presence of CD271-APC (ME20.4, 1:50, Cedarlane Laboratories) and MUC1 (115D8, 1:50, Monosan) followed by AF488 (IgG2b, 1:500, Life Technologies). Fibroblasts were incubated with CD105-AF488 (SN6, 1:25, AbD Serotec) and CD26 (202–36, 1:200, Abcam), followed by AF647 (IgG2b, 1:500). Controls were without primary antibody. 1 μg/ml propidium iodide (Invitrogen) or Fixable Viability Stain 780 (1:1000, BD Biosciences) was added 10 minutes prior to analysis and sorting (FACSAria I and II; BD Biosciences).

### Assessment of proliferation

CD105 (endoglin)^high^/CD26 (dipeptidyl peptidase-4)^low^ and CD105^low^/CD26^high^ cultures were split weekly at 5600 cells/cm^2^ and the number of population doublings were calculated as:

$$ \mathrm{n} = 3.32\left(\mathrm{Log}\ \mathrm{U}\mathrm{C}\mathrm{Y}\ \hbox{-}\ \mathrm{Log}\ \mathrm{I}\right) + \mathrm{X} $$

in which n = population doubling, UCY = cell yield, I = inoculum number, X = population doubling of inoculum).

Triplicate cultures seeded at 2770 cells/cm^2^ in passage 9 were used to quantify cell culture dynamics. Triplicate cultures representing three different biopsies were used to determine the endpoint number. Cells were counted manually using a Burker-Türk chamber.

### Co-culture

CD271^low^/MUC1^high^ primary luminal epithelial cells were seeded at 6000 cells/cm^2^ on confluent fibroblast feeders of CD105^high^/CD26^low^ and CD105^low^/CD26^high^ cells, respectively, in modified breastoid base medium without HEPES [9] (DMEM/F-12, 1:1), 1 μg/ml hydrocortisone (Sigma-Aldrich), 9 μg/ml insulin (Sigma-Aldrich), 5 μg/ml transferrin (Sigma-Aldrich), 5.2 ng/ml Na-Selenite (BD Industries), 100 μM ethanolamine (Sigma-Aldrich), 20 ng/ml basic fibroblast growth factor (PeproTech), 5 nM amphiregulin (R&D Systems), with the addition of 10 μM Y-27632 (Axon Medchem), 1.8 × 10^−4^ M adenine (Sigma Aldrich) and the serum replacement B27 (20 μl/ml, Life Technologies) [6]. hMSC feeders cultured under similar conditions were used for comparison.

To determine whether luminal progenitors and differentiated cells responded differently to co-culture, FACS-sorted CD166 (ALCAM)^high^/laminin receptor 67LR^high^ (EpCAM^high^/CD166^high^/LNR67^high^) or CD166^high^ (EpCAM^high^/CD90^low^/CD166^high^) differentiated luminal cells versus 67LR^low^ (EpCAM^high^/CD166^low^/LNR67^low^) or CD166^low^ (EpCAM^high^/CD90^low^/CD166^low^) progenitors [6] were confronted with either CD105^high^/CD26^low^ or CD105^low^/CD26^high^ cells under similar conditions. Epithelial structure formation was observed for up to three weeks by phase contrast microscopy and photographed (Leica DM IL).

### Immunostaining

Cryostat sections (6–8 μm) and cultures were stained by immunoperoxidase essentially as described, including incubation without primary antibody as control [5, 10, 11], for CD26 (1:50), CD105 (SN6, 1:200, Abcam) or MUC1 (1:100). Staining was photographed with the Leica DM5500B. For fluorescence staining, cryostat sections were incubated with CD26 (1:50) and CD105 (1:100) for 60 minutes, and AF568 (IgG2b, 1:500) and AF488 (IgG1, 1:500) for 30 minutes. Co-cultures were stained for fluorescence with MUC1 (1:10), K19 (Ba16, 1:50, Abcam) and K14 (LL002, 1:25, Abcam) followed by AF568 (IgG1, 1:500) and AF488 (IgG2b or IgG3, 1:500). Smooth muscle differentiation was detected by double staining for α-smooth muscle actin (1A4, 1:1000, Sigma) and CD105 (SN6, 1:50) followed by AF568 (IgG2a, 1:500) and AF488 (IgG1, 1:500). Fluorescence staining was evaluated and photographed using confocal microscopy (Zeiss LSM 700).

Images of co-cultures immunoperoxidase-stained for K19 (1:200) were acquired on a Leica Z6 AP0 at 1.25 magnification. A minimum of 2.2 cm^2^ was imaged and quantified (ImageJ, version 1.49 t). All images were analyzed in batch using a macro. Briefly, images were converted to 8-bit and duplicated. While one image was inverted and 160 was subtracted from it, the Differentials plugin [12] and the Laplacian operation was applied to the other followed by re-conversion to 8-bit. The two images were combined by multiplication and binarized by the Make Binary plugin. Images were analyzed by the Analyze Particles command using a lower size threshold of 0.0026 mm^2^ and excluding structures touching the image edge. Data were exported to Excel for plotting and handling.

### Microarray analysis

Total RNA was isolated in triplicate from CD105^high^/CD26^low^ and CD105^low^/CD26^high^ cells in passage 9 using the GenElute Mammalian Total RNA Miniprep Kit (Sigma-Aldrich): 100 ng RNA was labeled using the Ambion® WT Expression Kit (Ambion), and hybridized to the Affymetrix GeneChip®Human Gene 2.0 ST Array (Affymetrix) for 16 hours at 45 °C at 60 rpm and scanned with The GeneChip Scanner 3000 7G (Affymetrix). Data were imported into the GeneSpring GX 13.0 software and quantile-normalized with the RMA16 algorithm. For variance stabilization 16 was added before log2 transformation. Transcripts were annotated with netaffx annotation build 34. Statistical analyses of gene expression data were performed using the unpaired *t* test with Benjamini and Hochberg false discovery rate (FDR) *p* value correction. A total of 302 genes were considered significantly differentially expressed by a statistical significance threshold (corrected *p* < 0.05) and fold change larger than two. From these, 44 were selected for heat map presentation. A possible overlap among the 302 differentially expressed genes with previously published profiles of breast tumor versus normal stroma was analyzed using Oncomine.org., and tested for significance by Fisher’s exact test if common between at least two out of three studies [13–15].

### Analysis of differentiation potential

For adipogenic differentiation fibroblasts were seeded overnight at 40,000 cells/cm^2^ in 6-well plates (Nunc), and changed to adipogenic induction medium (AIM) [16]. Adipogenic induction was visualized by Oil Red O staining at day 15. Cultures were fixed for 10 minutes in 4 % paraformaldehyde, rinsed in 3 % isopropanol and stained with Oil Red O (Sigma-Aldrich, 25 mg Oil Red O dye, 5 ml 100 % isopropanol and 3.35 ml water) for one hour at room temperature. For osteogenic differentiation, fibroblasts were seeded overnight at 20,000 cells/cm^2^, and changed to osteoblastic induction medium (OIM) [16]. Controls were exposed to minimal essential medium (MEM, Invitrogen) with 10 % FBS.

For early osteogenic differentiation, alkaline phosphatase activity was measured and normalized to cell viability as described [17]. Alizarin Red staining was used to assess in vitro formation of mineralized matrix at day 15 after osteogenic induction as described [16]. Controls were exposed to MEM with 10 % FBS. Induction of differentiation in hMSCs was used as positive control in all assays.

Smooth muscle differentiation of CD105^high^/CD26^low^ cells was demonstrated by plating in passage 17 at a density of 4000 cells/cm^2^ on Primaria^TM^ in DMEM:F12 with glutamine followed by starvation for two days prior to exposure to 20 % serum for four days. High serum concentration has previously been shown to enhance α-smooth muscle actin in breast fibroblasts [18].

### RNA extraction and real-time quantitative polymerase chain reaction (RT-qPCR)

For analysis of gene expression levels of osteogenic and adipogenic markers in induced and non-induced cultures, total RNA was extracted using TRIzol and first strand cDNA was prepared by the revertAid H minus first-strand cDNA synthesis kit (Fermentas, St. Leon-Rot, Germany). RT-qPCR was performed using the StepOnePlus qPCR system and FAST SYBR Green master mix. Gene expression levels were determined using the formula 1/2ΔCT, in which ΔCT represents the difference between the target and the geometric mean of reference genes. Adhering to the guidelines for minimum information for publication of RT-qPCR experiments [19], we employed two reference genes for normalization: Beta-2-microglobulin (*β2m*) and ubiquitin C (*UBC*). Primers used are listed in Table 1.

**Table 1:** Primers for RT-qPCR analysis

Gene symbol	Forward primer	Reverse primer
*CEBPA*	AAC CTT GTG CCT TGG AAA TG	CTG TAG CCT CGG GAA GGA G
*Col1a1*	AGG GCT CCA ACG AGA TCG AGA TCC G	TAC AGG AAG CAG ACA GGG CCA ACG TCG
*Runx2*	TCT TCA CAA ATC CTC CCC	TGG ATT AAA AGG ACT TGG
*β2m*	CCT TGA GGC TAT CCA GCG T	CCT GCT CAG ATA CAT CAA ACA TG
*UBC*	ATT TGG GTC GCG GTT CTT G	TGC CTT GAC ATT CTC GAT GGT

To validate microarray results, RT-qPCR of 22 representative transcripts was performed in triplicate and further confirmed using RNA extracted from cells in passage 11 derived from another biopsy. Total RNA was reverse-transcribed using the High Capacity RNA-to-cDNA Kit (Applied Biosystems). RT-qPCR was performed as described [6] using TaqMan Gene Expression Assays (Applied Biosystems) and primers listed in Table 2. Glyceraldehyde-3-phosphate-dehydrogenase (*GAPDH*), Transferrin receptor (*TFRC*), hypoxanthine phosphoribosyltransferase 1 (*HPRT1*) and phosphoglycerate kinase 1 (*PGK1*) served as reference genes for normalization and gene expression levels were calculated by the formula 1/2ΔCT.

**Table 2:** TaqMan primers for microarray confirmation

Gene symbol	Assay ID	Gene symbol	Assay ID
*ENG (CD105)*	Hs00923996_m1	*SCUBE3*	Hs00738371_m1
*HGF*	Hs00300159_m1	*FNDC1*	Hs00287359_m1
*CFB*	Hs00156060_m1	*DPT*	Hs00355056_m1
*C3*	Hs00163811_m1	*GDF6*	Hs01377663_m1
*IL33*	Hs00369211_m1	*ACVRL1*	Hs00953798_m1
*COL4A1*	Hs00266237_m1	*ACVR2A*	Hs00155658_m1
*TNC*	Hs01115665_m1	*ACTA2*	Hs00426835_g1
*COL15A1*	s00266332_m1	*LAMA2*	Hs01124081_m1
*CNN1*	Hs00154543_m1	*TSPAN2*	Hs00194836_m1
*GPRC5B*	Hs00212116_m1	*HPRT1*	Hs99999909_m1
*IL1RL1*	Hs00249384_m1	*GAPDH*	Hs02758991_g1
*DCN*	Hs00370384_m1	*PGK1*	Hs00943178_g1
*COL11A1*	Hs01097664_m1	*TFRC*	Hs00951083_m1

## Results

**Fig. 1:**
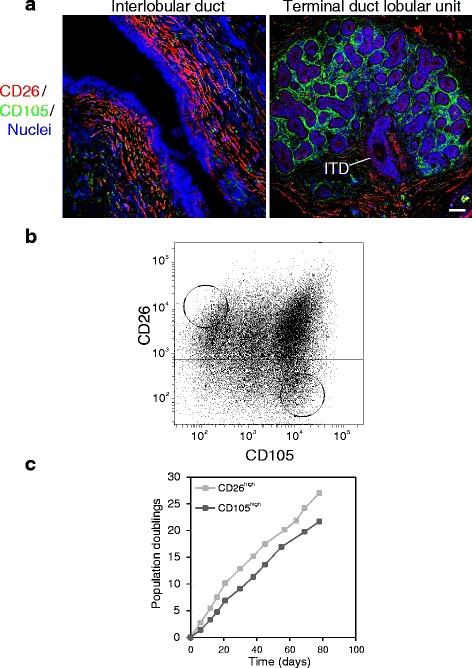
Characterization, isolation and cultivation of interlobular and intralobular fibroblasts. **a** Multicolor imaging of cryostat sections of normal breast tissue showing an interlobular duct (*left*) and a terminal duct lobular unit (TDLU, *right*) stained for CD105 (*green*), CD26 (*red*) and nuclei (*blue*). An intralobular terminal duct (*ITD*) connects the TDLU to the interlobular duct. Phenotypically distinct fibroblasts surround the two anatomical structures. **b** Fluorescence activated cell sorting diagram of serially passaged fibroblasts stained with CD26 and CD105. *Circles* indicate gates selected for sorting. **c** Time course of population doublings of CD26^high^ (*light squares*) and CD105^high^ (*dark squares*) fibroblasts in serial passage subculture (*scale bar* = 50 μm)

We and others have reported a stem cell zone in TDLUs of the human breast [5, 20]. To identify possible juxtaepithelial cells responsible for growth of luminal progenitors in TDLU we screened our antibody library with respect to relevant topographical fibroblast heterogeneity. Two surface markers, CD105 and CD26, consistently exhibited a non-overlapping staining pattern, one of which, CD26, has previously been shown to distinguish intralobular and interlobular breast stroma [21, 22]. In all specimens containing lobules (14 out of 17 biopsies), CD105, aside from staining the microvasculature, primarily stained stromal cells delineating acini, while CD26 was consistently present in the interlobular stroma albeit with varying intensity and in some instances (2 out of 17 biopsies) primarily surrounding interlobular ducts. In general, CD26-positive cells were completely absent from intralobular stroma except for occasional cells surrounding intralobular terminal ducts (Fig. 1a).

**Fig. 2:**
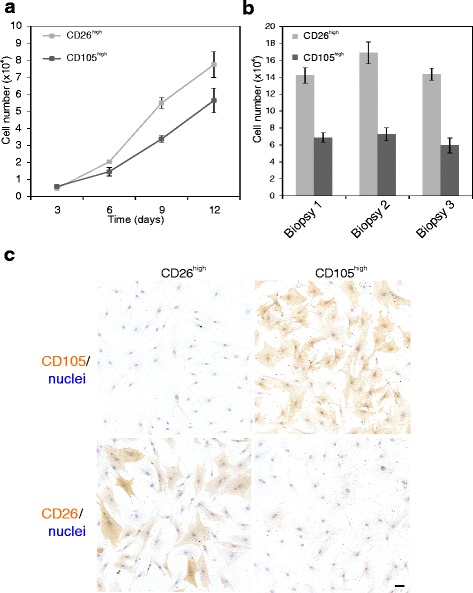
CD26^high^ and CD105^high^ fibroblasts are inherently different with respect to growth and staining pattern. **a** Representative growth curves of CD26^high^ (*light squares*) and CD105^high^ (*dark squares*) fibroblasts from eighth-passage cells in triplicate (*error bars* represent mean +/−SD). **b** Endpoint number at day 12 per 24-well in triplicate of CD26^high^ (*light bars*) and CD105^high^ (*dark bars*) fibroblasts from three different biopsies (*error bars* represent mean +/−SD). CD26^high^ fibroblasts consistently grow faster than CD105^high^ fibroblasts and reach a higher cell density at day 9 and 12, respectively (unpaired Student’s *t* test, *p* < 0.05). **c** Immunoperoxidase staining and nuclei counterstain in passage 9, showing that distinct phenotypes are passed on (*scale bar* = 50 μm)

In spite of some variance in FACS pattern among biopsies, CD105^high^/CD26^low^ and CD105^low^/CD26^high^ cells could be readily purified from 5/5 biopsies (Fig. 1b) into cell strains that could be propagated for more than 20 population doublings (Fig. 1c). The two phenotypes could be distinguished by immunocytochemical staining in primary culture, but to increase yield, fibroblasts were expanded prior to cell sorting. The two cell populations were inherently different with respect to growth rate; CD105^high^/CD26^low^ cells consistently growing slower than CD105^low^/CD26^high^ cells (Fig. 2a and b). The CD105^high/low^ phenotype was maintained with passage, while a differential expression of CD26 remained in 3/5 biopsies (Fig. 2c). Thus, we found CD105 to be a more reliable marker for distinguishing the two phenotypes. The cell strains could easily be passaged beyond passage 15.

**Fig. 3:**
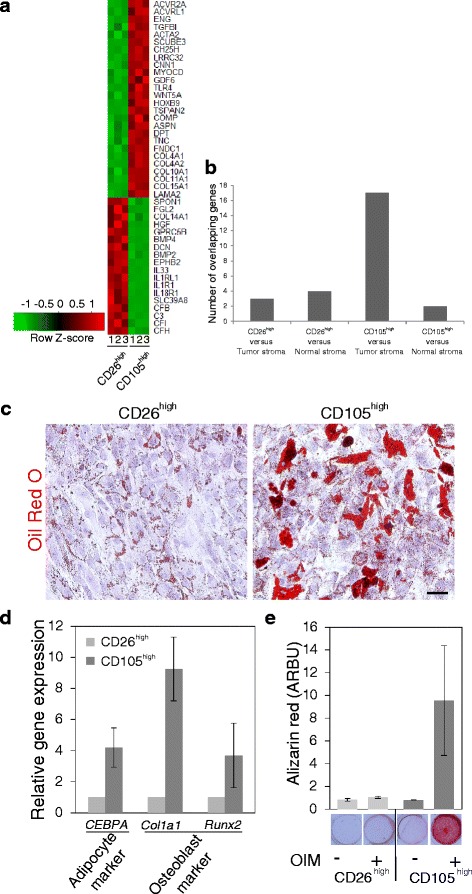
CD105^high^ fibroblasts exhibit a transforming growth factor (TGF)β profile and mesenchymal stem-like properties. **a** Heat map representation of microarray analysis of 44 selected, differentially expressed genes in CD26^high^ and CD105^high^ fibroblasts (passage 9). *Color key* indicates centered and row-scaled normalized intensity values. **b** Overlap between the top 302 median ranked significantly and differentially expressed genes and previously published profiles of breast normal and tumor stroma, respectively. *Bars* indicate the number of overlapping genes in CD26^high^ or CD105^high^ cells compared to tumor or normal stroma, respectively. The overlap between genes expressed by CD105^high^ cells and tumor stroma was statistically significant on analysis by Fisher’s exact test (*p* < 0.001). **c** Oil Red O staining of lipid droplets at day 15 of adipocyte differentiation in CD26^high^ and CD105^high^ cells, respectively, with nuclei stained with hematoxylin. **d** RT-qPCR of osteoblast and adipocyte marker gene expression (CCAAT/Enhancer binding protein, alpha (*CEBPA*), Collagen type 1 alpha 1 (*Col1a1*), and Runt-Related Transcription Factor 2 (*Runx2*)) in CD26^high^ (*light bars*) and CD105^high^ (*dark bars*) cells at day 3 of differentiation in passage 11 presented as gene expression relative to the geometric mean of two reference genes (*UBC* and *B2m*). The difference was statistically significant for *CEBPA* and *Col1a1* in three different biopsies on analysis by unpaired Student’s *t* test at *p* < 0.05. **e** Alizarin Red staining and quantification of the mineralized matrix at day 15 after exposure to osteogenic induction medium (*OIM*, +) or control conditions without inducing factors (−). On analysis by unpaired Student’s *t* test the difference in Alizarin Red staining in samples representing four biopsies, two in passage 11 and two in passage 13, was not statistically significant in CD26^high^ with and without osteogenic induction, but was significant at *p* < 0.05 in CD105^high^ with and without induction (*scale bar* = 100 μm). *Error bars* represent mean +/− SD

To investigate the characteristics of the two cell lineages in more detail, a microarray analysis was performed (Fig. 3a) and the observed differences were validated by RT-qPCR (Additional file 1: Figure S1) and confirmed in another biopsy. The molecular signature revealed that the profile of CD105^high^/CD26^low^ cells included induction of myofibroblast-related characteristics i.e. genes regulated by transforming growth factor-beta 1, such as *ACTA2*, *COLL*, *TNC* and *FNDC1* as compared to an immune-system-related signature, including complement factors, SCL39A8 [23], IL33/IL1RL1 [24], IL1R1 [25] and IL18R1 [26] in the CD105^low^/CD26^high^ cells (Fig. 3a). Differential expression of COL14A1/undulin is in accordance with findings by others [27].

The similarity between CD105^high^/CD26^low^ cells and myofibroblasts, including possible co-expression of CD105 and alpha-smooth muscle actin (Additional file 2: Figure S2), was further supported by comparison with previous datasets of differentially expressed genes in breast tumor versus normal stroma [13–15]. Thus, among genes differentially expressed between normal breast and breast cancer, the majority of genes in common with the present profiles were identified between CD105^high^ cells and tumor stroma (*p* < 0.001, Fig. 3b). The overlap between genes expressed by CD105^high^ cells and tumor stroma included Wingless-type MMTV integration site family member 5A (*WNT5A*), Vitamin D (1,25- dihydroxyvitamin D3) receptor (*VDR*), Sulfatase 2 (*SULF2*), Sushi repeat containing protein x-linked 2 (*SRPX2*), Secreted frizzled-related protein 2 (*SFRP2*), Phospholipid phosphatase 4 (*PLPP4*), NADPH oxidase 4 (*NOX4*), Leucine rich repeating containing 15 (*LRRC15*), Lim and cysteine rich domains 1 (*LMCD1*), Interferon gamma-inducible protein 30 (*IFI30*), Growth arrest and DNA-damage inducible beta (*GADD45*B), Fibronectin type III domain containing 1 (*FNDC1*), Fc Fragment of IgE receptor Ig (*FCER1G*), Cartilage oligomeric matrix protein (*COMP*), Collagen type XI alpha 1 (*COL11A1*), Collagen type X alpha 1 (*COL10A1*), and Asporin (*ASPN*).

As CD105 has been identified as a marker of MSCs [28], we determined the differentiation capacity of the two populations as compared to hMSCs. Only the CD105^high^/CD26^low^ cells resembled hMSCs by the potential to differentiate along adipogenic and osteogenic lineages (Fig. 3c and d and Additional file 3: Figure S3), and this difference in response was maintained up to passage 15 (Additional file 3: Figure S3).

**Fig. 4:**
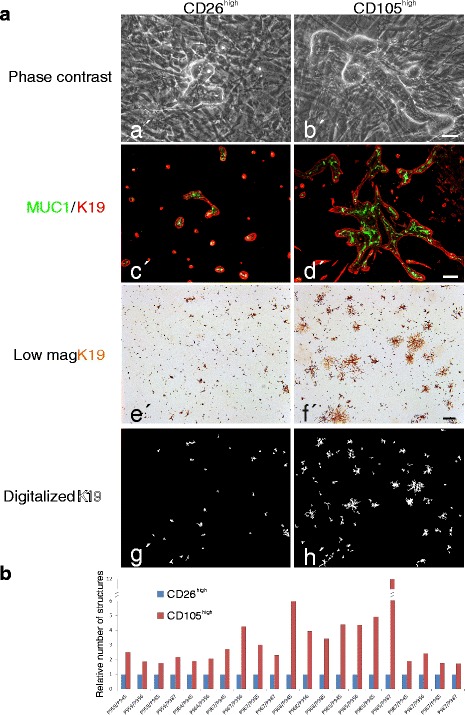
CD105^high^ stroma is a specialized microenvironment for branching morphogenesis of luminal breast epithelial cells. **a** Primary cultures of purified luminal breast epithelial cells plated at clonal density on confluent feeders of CD26^high^ (*left column*) or CD105^high^ (*right colum*n) fibroblasts: phase contrast micrographs of co-cultures twenty days after plating showing branching morphogenesis primarily on CD105^high^ fibroblasts (*a*’, *b*’); dual-color imaging of co-cultures stained with keratin K19 (red) and MUC1 (*green*). Note the correctly polarized staining pattern and the elaborate structures on CD105^high^ fibroblasts (*c*’, *d*’); low-magnification micrographs of co-cultures ten days after plating and immunoperoxidase staining for keratin K19 (*e*’, *f*’); digitalized images of keratin K19-stained epithelial structures (*g*’, *h*’). **b** Quantitative representation of K19-stained morphological structures in multiple recombinant cultures representing eight biopsies showing consistent growth advantage of luminal epithelial cells on CD105^high^ fibroblasts (*red bars*) as normalized in each set of samples to structures formed on CD26^high^ fibroblasts (*blue bars*) (*scale bar* = 50 μm (*a*’, *b*’); 100 μm (*c*’, *d*’); 500 μm (*e*’-*h*’))

To test whether stromal cells are functionally imprinted by their site of origin, we next investigated the association between luminal breast epithelial growth and the CD105/CD26 lineages. Seeding of single cell CD271^low^/MUC1^high^ luminal epithelial cells (Additional file 4: Figure S4) on fibroblast feeders resulted in the formation of tubular structures with a central lumen within three weeks (Fig. 4a). Staining with epithelial lineage markers MUC1, keratin K19 and keratin K14 revealed that the structures were indeed luminal and in addition were correctly polarized (Fig. 4a, c’-d’). Moreover, it was evident that the structures expanded much more on CD105^high^/CD26^low^ than on CD105^low^/CD26^high^ fibroblasts (Fig. 4a). Quantitative image analysis revealed a consistently higher level of branching morphogenesis on lobular, CD105^high^/CD26^low^ fibroblasts, compared to interlobular, CD105^low^/CD26^high^ fibroblasts in all combinations of biopsies tested (Fig. 4b).

The relevance of the tissue of origin of CD105^high^ cells was further supported by the finding that CD105^+^ hMSCs did not support tubular structure formation. These experiments suggested that crude epithelial populations contain progenitors that are able to respond to a relevant microenvironment upon appropriate stimulation. This was confirmed by confronting purified epithelial progenitors and differentiated epithelial cells, respectively [6], with CD105^low^/CD26^high^ and CD105^high^/CD26^low^ fibroblasts. While differentiated epithelial cells remained as single cells, purified progenitors proliferated and generated correctly polarized structures (Additional file 5: Figure S5). Taken together, these results are in strong favor of the existence of two lineages of stromal cells in the human breast, one of which has characteristics in common with MSCs and provides a specialized microenvironment for luminal progenitors in the TDLU.

## Discussion and conclusions

Previous attempts to demonstrate stable fibroblast lineages from the normal human breast by serial passage have been affected by phenotypic drifting of the isolated cells [22]. Early-passage crudely isolated intralobular fibroblasts were devoid of CD26 (DPPIV), but with passage they had CD26 induced and became indistinguishable from interlobular fibroblasts [22].

In the present study, intralobular fibroblasts are positively identified and isolated based on a high expression of CD105, and we found that the CD105^high/low^ phenotype is stably maintained with extended culture. We therefore suggest that CD105 is a more reliable marker for distinguishing intralobular and interlobular fibroblasts. Moreover, cellular and molecular analyses were employed to establish whether the two populations isolated in the present study indeed remain functionally different. We demonstrated that CD105^high^/CD26^low^ lobular fibroblasts and CD105^low^/CD26^high^ interlobular fibroblasts represent two distinct functionally specialized lineages. Both lineages grow in culture and maintain their CD105/CD26 phenotype. However, the CD105^high^/CD26^low^ lobular fibroblasts are further distinguished by their capacity to differentiate into adipogenic and osteogenic lineages. Moreover, upon exposure to serum originally shown to reveal myofibroblastic differentiation in normal breast fibroblasts [18], the gene expression profile of CD105^high^/CD26^low^ cells partly overlaps with the profile of breast tumor stroma. This might indicate that intralobular fibroblasts are more prone to generating myofibroblasts should cancer arise in the TDLU - the predominant site of breast tumor occurrence *per se*.

That functional heterogeneity within a tissue stromal compartment may exist has been described by others. Thus, in mouse skin, two subpopulations of fibroblasts derived from a common fibroblast progenitor localize to the upper and lower dermis, respectively [2]. Interestingly, like the two fibroblast lineages described here, one expresses CD26 and the other is capable of undergoing adipogenic differentiation suggesting that these characteristics may serve as more general markers of stromal cell type stratification [2, 29]. Furthermore, heterogeneity amongst fibroblasts and distinct fibroblast features has been implicated as a risk factor for developing breast cancer. Of note, a more migratory fibroblast phenotype has been detected in relatives of patients with hereditary breast cancer [30] (reviewed in [31]), emphasizing that more knowledge of the stromal compartment in general may be relevant for the understanding of cancer pathogenesis.

Several independent observations point towards luminal progenitors as potential precursors of human breast cancer, somewhat surprisingly also including basal-like breast cancer [32, 33]. This has put an enormous emphasis on luminal cells in developing reliable assays for human breast morphogenesis and homeostasis not least from the point of view that the lifetime risk of developing cancer correlates with the total number of divisions of long-lived cells in a tissue [34]. The co-culture assay presented here may prove suitable for such analyses. Specific populations of epithelial cells isolated as single cells can be plated on fibroblast feeders and the result of epithelial-stromal interaction can be monitored directly. The relevance of directly exposing luminal cells to stromal fibroblasts from which they *in situ* are separated by myoepithelial cells and basement membrane could be questioned. However, while luminal cells at a glance may seem completely enveloped by myoepithelial cells, higher magnifications reveal that they project into the surrounding stroma containing delimiting fibroblasts [35]. The interstitial stroma in turn forms a barrier between capillaries and epithelium, across which epitheliotrophic stimuli from the blood supply must pass [36]. The details of how this signaling and how the communication between epithelium and stromal cells across the stroma takes place remain to be unraveled.

Our data nevertheless favor that luminal epithelial progenitors receive input from the lobular stromal microenvironment. Thus, they respond by clonal expansion and branching morphogenesis, including the formation of correctly polarized luminal epithelial cells. It cannot be excluded that the effect of intralobular fibroblasts reported here in the adult breast may reflect a similar function in early development. In the infant breast, fibroblasts surrounding developing epithelial structures are devoid of CD26 (DPPIV) and thus, distinct from interlobular CD26 (DPPIV)-positive fibroblasts [37]. We further demonstrate that morphogenesis can occur independently of the presence of myoepithelial cells. Interestingly, in vivo where stroma is amply present, lineage tracing in the mouse mammary gland shows that the luminal epithelial compartment expands exclusively by self-duplication [38]. By contrast, in culture deprived of stroma, luminal epithelial cells apparently become multipotent, and those from mice in addition acquire mammary repopulating capability [10, 39, 40]. Thus, the present approach mimics the lobular epithelial microenvironment and unravels the activity of luminal epithelial progenitors. This finding may pave the way for further interrogation of developmental processes reflecting epithelial-stromal crosstalk in the normal human breast as well as in breast cancer.

## Additional files

Additional file 1: Figure S1. Confirmation of microarray analysis by RT-qPCR. RT-qPCR of a representative subset of differentially expressed genes between CD105^high^ and CD26^high^ fibroblasts presented as the relative normalized expression level in CD105^high^ to CD26^high^ fibroblasts. The genes analyzed include signal peptide, CUB domain and EGF like domain containing 3 (*SCUBE3*), CD105/endoglin (*ENG*), growth differentiation factor 6 (*GDF6*), collagen type XI alpha 1 chain (*COL11A1*), tetraspanin 2 (*TSPAN2*), collagen type IV alpha 1 chain (*COL4A1*), actin, alpha 2, smooth muscle, aorta (*ACTA2*), tenascin C (*TNC*), activin A receptor like type 1 (*ACVRL1*), collagen type XV alpha 1 chain (*COL15A1*), calponin 1 (*CNN1*), dermatopontin (*DPT*), fibronectin type III domain containing 1 (*FNDC1*), activin A receptor type 2A (*ACVR2A*), laminin subunit alpha 2 (*LAMA2*), interleukin 1 receptor like 1 (*IL1RL1*), interleukin 33 (*IL33*), hepatocyte growth factor (*HGF*), complement factor B (*CFB*); G protein-coupled receptor class C group 5 member B (*GPRC5B*), complement component 3 (*C3*) and decorin (*DCN*). *Error bars* represent mean +/− SD. (PDF 34 kb)
Additional file 2: Figure S2. Co-expression of CD105 and α-smooth muscle actin in CD105^high^ fibroblasts. Upon serum starvation and subsequent stimulation with 20 % serum, α-smooth muscle actin is further induced in CD105-expressing cells. Highly smooth muscle- differentiated cells tend to exhibit lower CD105 expression (*scale bar* = 50 μm). (PDF 1786 kb)
Additional file 3: Figure S3. CD105^high^ and CD26^high^ fibroblasts remain phenotypically and functionally different in high passage cultures. **a** Alkaline phosphatase (ALP) activity, an early marker of osteoblast differentiation, in osteogenic induced cultures (*light bars*) versus non-induced cultures (*dark bars*) of CD26^high^ and CD105^high^ cells, analyzed at day 6 after induction of three different biopsies (one in passage 9 and two in passage 10). The difference in ALP activity was significant on analysis by unpaired Student’s *t* test in induced versus non-induced CD105^high^ cells only (*p* < 0.0001). *Error bars* represent mean +/− SD. **b** ALP activity in osteogenic induced (*light bars*) versus non-induced (*dark bars*) cultures of CD26^high^ and CD105^high^ cells, analyzed at day 6 in passage 10 and 15, respectively, after initial sorting in passage 5. Data represent quadruplicate samples from two biopsies and are presented as arbitrary units (*ARBU*). CD105^high^ cells maintain their osteogenic differentiation capacity up to passage 15 indicating that their distinct functional properties are maintained in higher passages. *Error bars* represent mean +/− SD. (PDF 33 kb)
Additional file 4: Figure S4. Gating strategy to isolate uncultured primary breast MUC1^high^ epithelial cells by FACS. Uncultured primary breast cells from trypsinized organoids were incubated with antibodies against CD271, a marker of myoepithelial cells, and MUC1, a marker of luminal epithelial cells, and analyzed by FACS. The MUC1^high^ cells were selected and isolated as indicated. (PDF 30 kb)
Additional file 5: Figure S5. Branching morphogenesis reflects activity of luminal progenitors. Primary cultures of purified luminal breast epithelial cells plated at clonal density on confluent feeders of (*left column*) CD26^high^ or (*right column*) CD105^high^ fibroblasts stained for MUC1 by immunoperoxidase. Nuclear counterstain is omitted to clearly outline epithelial cells. EpCAM^high^/CD166^high^/67LR^high^ differentiated luminal epithelial cells (CD166^high^/67LR^high^) remained as single cells upon confrontation with fibroblast feeders (*upper panel*), while EpCAM^high^/CD166^low^/67LR^low^ progenitors (CD166^low^/67LR^low^) responded by undergoing branching morphogenesis with larger structures forming on CD105^high^ fibroblasts (*lower panel*) (*scale bar* = 100 μm). (PDF 9484 kb)

## Abbreviations

AIM: adipogenic induction medium
DMEM: Dulbecco’s modified Eagle’s medium
FACS: fluorescence activated cell sorting
FBS: fetal bovine serum
hMSC: human mesenchymal stem cells
MEM: minimal essential medium
OIM: osteogenic induction medium
RT-qPCR: real-time quantitative PCR
TDLU: terminal duct lobular unit

## References

[1] Ball EMA, Risbridger GP (2001). Activins as regulators of branching morphogenesis. Dev Biol.

[2] Driskell RR, Lichtenberger BM, Hoste E, Kretzschmar K, Simons BD, Charalambous M, Ferrori SR, Herault Y, Pavlovic G, Ferguson-Smith AC (2013). Distinct fibroblast lineages determine dermal architecture in skin development and repair. Nature.

[3] Rønnov-Jessen L, Petersen OW, Bissell MJ (1996). Cellular changes involved in conversion of normal to malignant breast: The importance of the stromal reaction. Physiol Rev.

[4] Cardiff RD, Wellings SR (1999). The comparative pathology of human and mouse mammary glands. J Mammary Gland Biol Neoplasia.

[5] Villadsen R, Fridriksdottir AJ, Rønnov-Jessen L, Gudjonsson T, Rank F, LaBarge MA, Bissell MJ, Petersen OW (2007). Evidence of a stem cell hierarchy in the adult human breast. J Cell Biol.

[6] Fridriksdottir AJ, Kim J, Villadsen R, Klitgaard MC, Petersen OW, Rønnov-Jessen L (2015). Propagation of oestrogen receptor-positive and oestrogen receptor-responsive normal human breast cells in culture. Nat Commun.

[7] Rønnov-Jessen L, Petersen OW (1993). Induction of α-smooth muscle actin by transforming growth factor-β1 in quiescent human breast gland fibroblasts. Implications for myofibroblast generation in breast neoplasia. Lab Invest.

[8] Simonsen JL, Rosada C, Serakinci N, Justesen J, Stenderup K, Rattan SIS, Jensen TG, Kassem M (2002). Telomerase expression extends the proliferative life-span and maintains the osteogenic potential of human bone marrow stromal cells. Nat Biotechnol.

[9] Pasic L, Eisinger-Mathason TSK, Velayudhan BT, Moskaluk CA, Brenin DR, Macara IG, Lannigan DA (2011). Sustained activation of the HER1-ERK1/2-RSK signaling pathway controls myoepithelial cell fate in human mammary tissue. Genes Dev.

[10] Petersen OW, van Deurs B (1988). Growth factor control of myoepithelial-cell differentiation in cultures of human mammary gland. Differentiation.

[11] Rønnov-Jessen L, Celis JE, van Deurs B, Petersen OW (1992). A fibroblast-associated antigen: Characterization in fibroblasts and immunoreactivity in smooth muscle differentiated stromal cells. J Histochem Cytochem.

[12] Thévenaz P, Unser M (2000). Optimization of mutual information for multiresolution image registration. IEEE Transact Image Process.

[13] Karnoub AE, Dash AB, Vo AP, Sullivan A, Brooks MW, Bell GW, Richardson AL, Polyak K, Tubo R, Weinberg RA (2007). Mesenchymal stem cells within tumour stroma promote breast cancer metastasis. Nature.

[14] Finak G, Bertos N, Pepin F, Sadekova S, Souleimanova M, Zhao H, Chen H, Omeroglu G, Meterissian S, Omeroglu A (2008). Stromal gene expression predicts clinical outcome in breast cancer. Nat Med.

[15] Ma X-J, Dahiya S, Richardson E, Erlander M, Sgroi DC (2009). Gene expression profiling of the tumor microenvironment during breast cancer progression. Breast Cancer Res.

[16] Jafari A, Siersbaek MS, Chen L, Qanie D, Zaher W, Abdallah BM, Kassem M (2015). Pharmacological inhibition of protein kinase G1 enhances bone formation by human skeletal stem cells through activation of RhoA-Aky signaling. Stem Cells.

[17] Qiu W, Hu Y, Andersen TE, Jafari A, Li N, Chen W, Kassem M (2010). Tumor necrosis factor receptor superfamily member 19 (*TNFRSR19*) regulates differentiation fate of human mesenchymal (stromal) stem cells through canonical Wnt signaling and C/EBP. J Biol Chem.

[18] Rønnov-Jessen L, van Deurs B, Celis JE, Petersen OW (1990). Smooth muscle differentiation in cultured human breast gland stromal cells. Lab Invest.

[19] Bustin SA, Benes V, Garson JA, Hellemans J, Huggett J, Kubista M, Mueller R, Nolan T, Pfaffi MW, Shipley GL (2009). The MIQE guidelines: minimum information for publication of quantitative real-time PCR experiments. Clin Chem.

[20] Honeth G, Schiavinotto T, Vaggi F, Marlow R, Kanno T, Shinomiya I, Lombardi S, Buchupalli B, Graham RA, Gazinska P (2015). Models of breast morphogenesis based on localization of stem cells in the developing mammary lobule. Stem Cell Rep.

[21] Atherton AJ, Monaghan P, Warburton MJ, Robertson D, Kenny AJ, Gusterson BA (1992). Dipeptidyl peptidase IV expression identifies a functional sub-population of breast fibroblasts. Int J Cancer.

[22] Atherton AJ, O'Hare MJ, Buluwela L, Titley J, Monaghan P, Paterson HF, Warburton MJ, Gusterson BA (1994). Ectoenzyme regulation by phenotypically distinct fibroblast sub-populations isolated from the human mammary gland. J Cell Sci.

[23] Liu M-J, Bao S, Gálvez-Peralta M, Pyle CJ, Rudawsky AC, Pavlovicz RE, Killilea DW, Li C, Nebert DW, Wewers MD (2013). The zinc transporter SLC39A8 is a negative feedback regulator of NF-κB through zinc-mediated inhibition of IKK. Cell Rep.

[24] Schmitz J, Owyang A, Oldham E, Song Y, Murphy E, McClanahan TK, Zurawski G, Moshrefi M, Qin J, Li X (2005). IL-33, and interleukin-1-like cytokine that signals via the IL-1 receptor-related protein ST2 and induces T helper type 2-associated cytokines. Immunity.

[25] Uhl J, Newton RC, Giri JG, Sandlin G, Horuk R (1989). Identification of IL-1 receptors on human monocytes. J Immunol.

[26] Sareneva T, Julkunen I, Matikainen S (2000). IFN-α and IL-12 induce IL-18 receptor gene expression in human NK and T cells. J Immunol.

[27] Atherton AJ, Warburton MJ, O'Hare MJ, Monaghan P, Schuppan D, Gusterson BA (1998). Differential expression of type XIV collagen/undulin by human mammary gland intralobular and interlobular fibroblasts. Cell Tissue Res.

[28] Barry FP, Boynton RE, Haynesworth S, Murphy JM, Zaia J (1999). The monoclonal antibody SH-2, raised against human mesenchymal stem cells, recognizes and epitope on endoglin (CD105). Biochem Biophys Res Commun.

[29] Driskell RR, Watt FM. Understanding fibroblast heterogeneity in the skin. Trends Cell Biol. 2015;25. 10.1016/j.tcb.2014.10.001 25455110

[30] Haggie JA, Sellwood RA, Howell A, Birch JM, Schor SL (1987). Fibroblasts from relatives of patients with hereditary breast cancer show fetal-like behaviour in vitro. Lancet.

[31] Schor SL, Schor AM (2001). Tumour-stroma interactions. Phenotypic and genetic alterations in mammary stroma: Implications for tumour progression. Breast Cancer Res.

[32] Lim E, Vaillant F, Wu D, Forrest NC, Pal B, Hart AH, Asselin-Labat ML, Gyorki DE, Ward T, Partanen A (2009). Aberrant luminal progenitors as the candidate target population for basal tumor development in *BRCA1* mutation carriers. Nat Med.

[33] Keller PJ, Arendt LM, Skibinski A, Logvinenko T, Klebba I, Dong S, Smith AL, Prat A, Perou CM, Gilmore H (2012). Defining the cellular precursors to human breast cancer. Proc Natl Acad Sci U S A.

[34] Tomasetti C, Vogelstein B (2015). Variation in cancer risk among tissues can be explained by the number of stem cell divisions. Science.

[35] Ozzello L (1970). Epithelial-stromal junction of normal and dysplastic mammary glands. Cancer.

[36] Eyden BP, Watson RJ, Harris M, Howell A (1986). Intralobular stromal fibroblasts in the resting human mammary gland: ultrastructural properties and intercellular relationships. J Submicrosc Cytol.

[37] Atherton AJ, Anbazhagan R, Monaghan P, Bartek J, Gusterson BA (1994). Immunolocalisation of cell surface peptidases in the developing human breast. Differentiation.

[38] Van Keymeulen A, Rocha AS, Ousset M, Beck B, Bouvencourt G, Rock J, Sharma N, Dekoninck S, Blanpain C (2011). Distinct stem cells contribute to mammary gland development and maintenance. Nature.

[39] Péchoux C, Gudjonsson T, Rønnov-Jessen L, Bissell MJ, Petersen OW (1999). Human mammary luminal epithelial cells contain progenitors to myoepithelial cells. Dev Biol.

[40] Makarem M, Kannan N, Nguyen LV, Knapp DJHF, Balani S, Prater MD, Stingl J, Raouf A, Nemirovsky O, Eirew P (2013). Developmental changes in the *in vitro* activated regenerative activity of primitive mammary epithelial cells. PLoS Biol.

