# Supplementary figures and images for: Evidence of two distinct functionally specialized fibroblast lineages in breast stroma
Source: Breast Cancer Res. 2016 Nov 3;18:108. doi: 10.1186/s13058-016-0769-2 (PMC5093959; doi:10.1186/s13058-016-0769-2)

**Figure S1. Confirmation of microarray analysis by RT-qPCR**

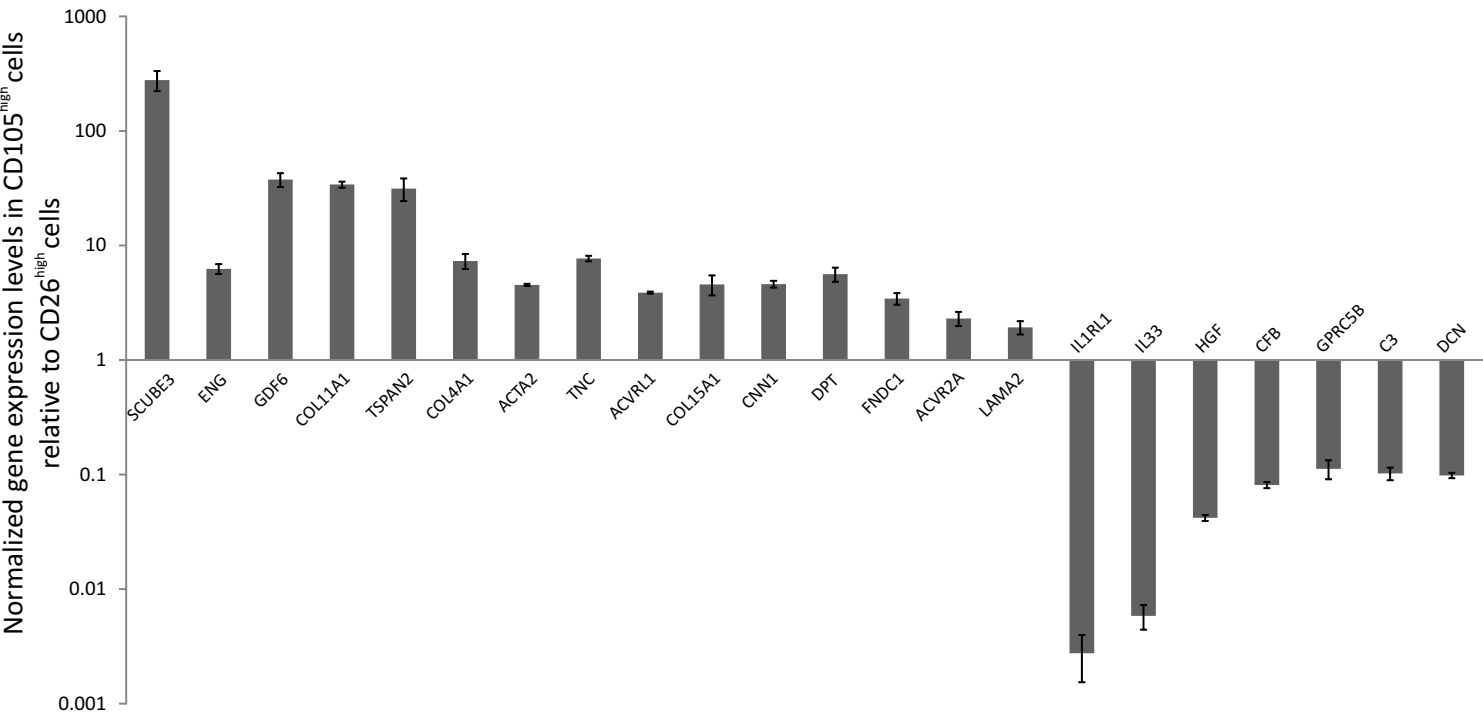

Supplement: Additional file 1: Figure S1. — Confirmation of microarray analysis by RT-qPCR. RT-qPCR of a representative subset of differentially expressed genes between CD105high and CD26high fibroblasts presented as the relative normalized expression level in CD105high to CD26high fibroblasts. The genes analyzed include signal peptide, CUB domain and EGF like domain containing 3 (SCUBE3), CD105/endoglin (ENG), growth differentiation factor 6 (GDF6), collagen type XI alpha 1 chain (COL11A1), tetraspanin 2 (TSPAN2), collagen type IV alpha 1 chain (COL4A1), actin, alpha 2, smooth muscle, aorta (ACTA2), tenascin C (TNC), activin A receptor like type 1 (ACVRL1), collagen type XV alpha 1 chain (COL15A1), calponin 1 (CNN1), dermatopontin (DPT), fibronectin type III domain containing 1 (FNDC1), activin A receptor type 2A (ACVR2A), laminin subunit alpha 2 (LAMA2), interleukin 1 receptor like 1 (IL1RL1), interleukin 33 (IL33), hepatocyte growth factor (HGF), complement factor B (CFB); G protein-coupled receptor class C group 5 member B (GPRC5B), complement component 3 (C3) and decorin (DCN). Error bars represent mean +/− SD. (PDF 34 kb) [file 13058_2016_769_MOESM1_ESM.pdf]

**Figure S2. Co-expression of CD105 and  $\alpha$ -smooth muscle actin in  $CD105^{high}$  fibroblasts**

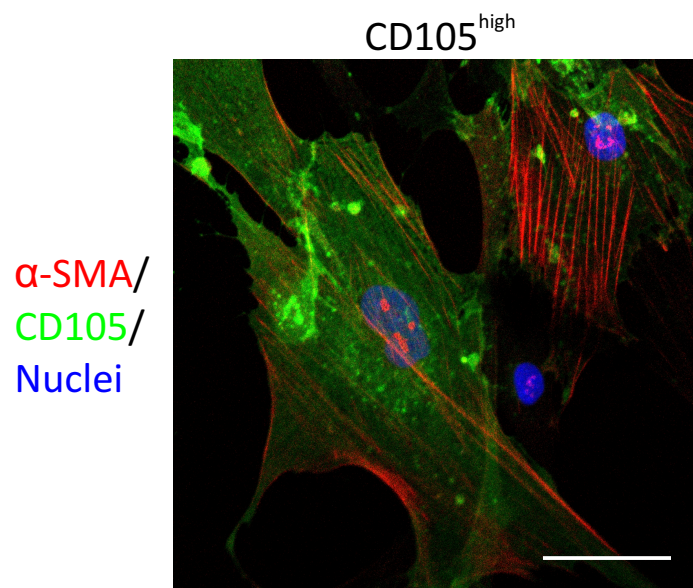

Supplement: Additional file 2: Figure S2. — Co-expression of CD105 and α-smooth muscle actin in CD105high fibroblasts. Upon serum starvation and subsequent stimulation with 20 % serum, α-smooth muscle actin is further induced in CD105-expressing cells. Highly smooth muscle- differentiated cells tend to exhibit lower CD105 expression (scale bar = 50 μm). (PDF 1786 kb) [file 13058_2016_769_MOESM2_ESM.pdf]

**Figure S5. Branching morphogenesis reflects activity of luminal progenitors**

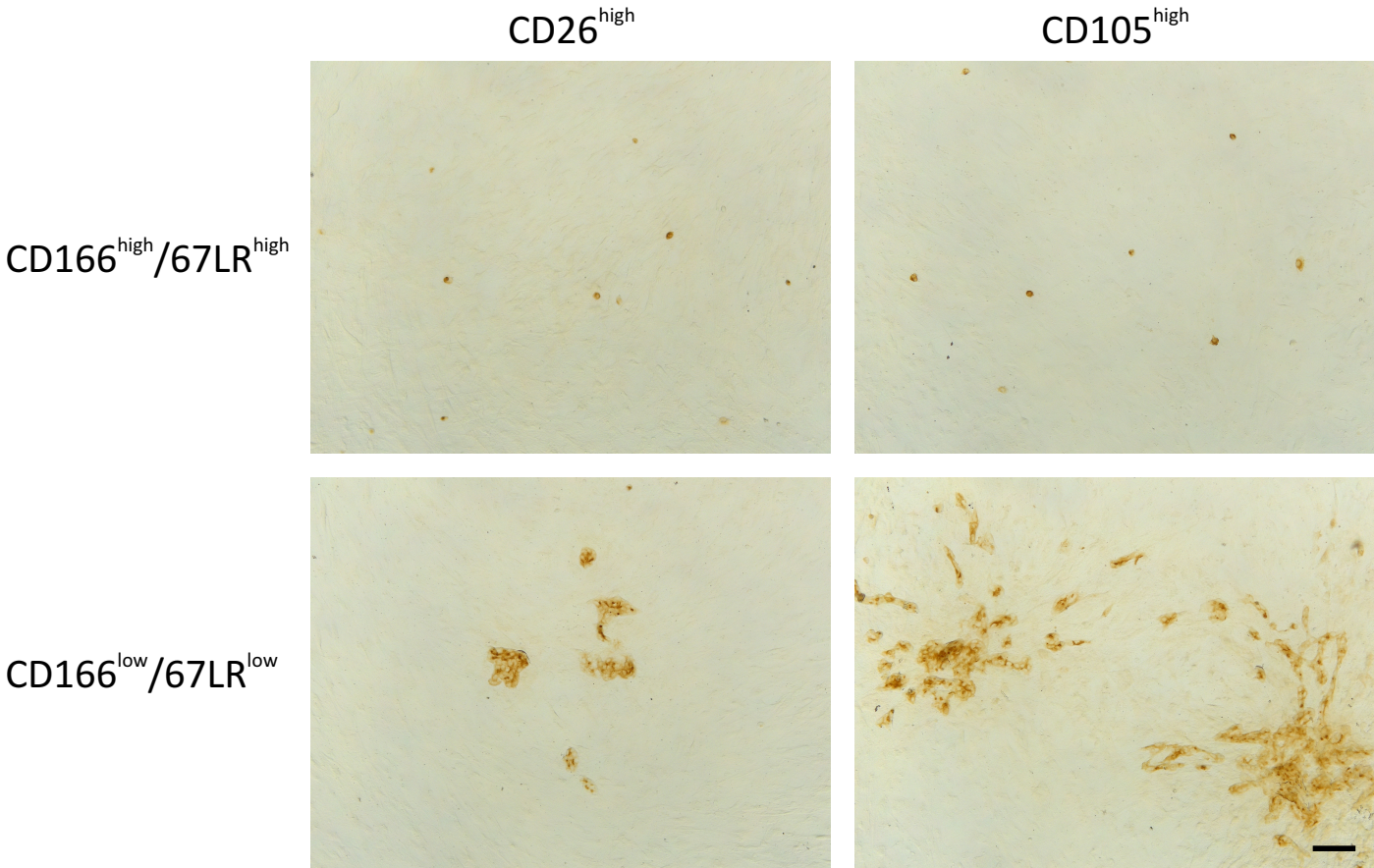

Supplement: Additional file 5: Figure S5. — Branching morphogenesis reflects activity of luminal progenitors. Primary cultures of purified luminal breast epithelial cells plated at clonal density on confluent feeders of (left column) CD26high or (right column) CD105high fibroblasts stained for MUC1 by immunoperoxidase. Nuclear counterstain is omitted to clearly outline epithelial cells. EpCAMhigh/CD166high/67LRhigh differentiated luminal epithelial cells (CD166high/67LRhigh) remained as single cells upon confrontation with fibroblast feeders (upper panel), while EpCAMhigh/CD166low/67LRlow progenitors (CD166low/67LRlow) responded by undergoing branching morphogenesis with larger structures forming on CD105high fibroblasts (lower panel) (scale bar = 100 μm). (PDF 9484 kb) [file 13058_2016_769_MOESM5_ESM.pdf]
