# Supplementary material for: Evidence of two distinct functionally specialized fibroblast lineages in breast stroma
Source: Breast Cancer Res. 2016 Nov 3;18:108. doi: 10.1186/s13058-016-0769-2 (PMC5093959; doi:10.1186/s13058-016-0769-2)

Figure S3. CD105[high] and CD26[high] fibroblasts remain phenotypically and functionally different in high passage cultures

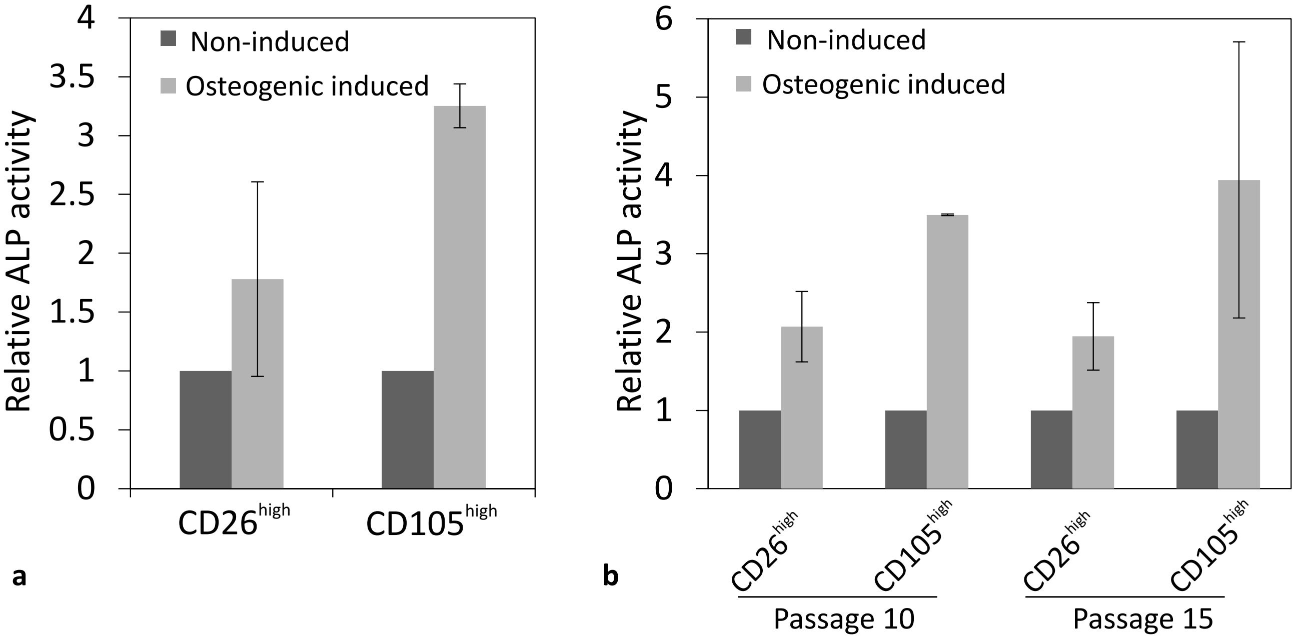

Supplement: Additional file 3: Figure S3. — CD105high and CD26high fibroblasts remain phenotypically and functionally different in high passage cultures. a Alkaline phosphatase (ALP) activity, an early marker of osteoblast differentiation, in osteogenic induced cultures (light bars) versus non-induced cultures (dark bars) of CD26high and CD105high cells, analyzed at day 6 after induction of three different biopsies (one in passage 9 and two in passage 10). The difference in ALP activity was significant on analysis by unpaired Student’s t test in induced versus non-induced CD105high cells only (p < 0.0001). Error bars represent mean +/− SD. b ALP activity in osteogenic induced (light bars) versus non-induced (dark bars) cultures of CD26high and CD105high cells, analyzed at day 6 in passage 10 and 15, respectively, after initial sorting in passage 5. Data represent quadruplicate samples from two biopsies and are presented as arbitrary units (ARBU). CD105high cells maintain their osteogenic differentiation capacity up to passage 15 indicating that their distinct functional properties are maintained in higher passages. Error bars represent mean +/− SD. (PDF 33 kb) [file 13058_2016_769_MOESM3_ESM.pdf]
