# Supplementary material for: Evidence of two distinct functionally specialized fibroblast lineages in breast stroma
Source: Breast Cancer Res. 2016 Nov 3;18:108. doi: 10.1186/s13058-016-0769-2 (PMC5093959; doi:10.1186/s13058-016-0769-2)

Figure S4. Gating strategy to isolate uncultured primary breast MUC1^{high} epithelial cells by FACS

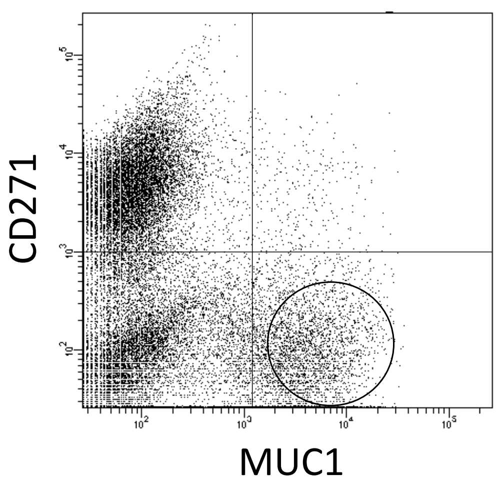

Supplement: Additional file 4: Figure S4. — Gating strategy to isolate uncultured primary breast MUC1high epithelial cells by FACS. Uncultured primary breast cells from trypsinized organoids were incubated with antibodies against CD271, a marker of myoepithelial cells, and MUC1, a marker of luminal epithelial cells, and analyzed by FACS. The MUC1high cells were selected and isolated as indicated. (PDF 30 kb) [file 13058_2016_769_MOESM4_ESM.pdf]
